# Aflatoxin B1 Induces Neurotoxicity through Reactive Oxygen Species Generation, DNA Damage, Apoptosis, and S-Phase Cell Cycle Arrest

**DOI:** 10.3390/ijms21186517

**Published:** 2020-09-06

**Authors:** Boyan Huang, Qingmei Chen, Lingling Wang, Xiaojuan Gao, Wenya Zhu, Peiqiang Mu, Yiqun Deng

**Affiliations:** 1 Guangdong Provincial Key Laboratory of Protein Function and Regulation in Agricultural Organisms, College of Life Sciences, South China Agricultural University, Tianhe District, Guangzhou 510642, China; huangboyan123@stu.scau.edu.cn (B.H.); chenqingmei@scau.edu.cn (Q.C.); llwang417@scau.edu.cn (L.W.); gaoxiaojuan@stu.scau.edu.cn (X.G.); zhuwenya@stu.scau.edu.cn (W.Z.); 2 Key Laboratory of Zoonosis of Ministry of Agriculture and Rural Affairs, South China Agricultural University, Guangzhou 510642, China

**Keywords:** aflatoxin B1, neurotoxicity, reactive oxygen species, DNA damage, cell cycle arrest, apoptosis

## Abstract

Aflatoxin B1 (AFB_1_) is a mycotoxin widely distributed in a variety of food commodities and exhibits strong toxicity toward multiple tissues and organs. However, little is known about its neurotoxicity and the associated mechanism. In this study, we observed that brain integrity was markedly damaged in mice after intragastric administration of AFB_1_ (300 μg/kg/day for 30 days). The toxicity of AFB_1_ on neuronal cells and the underlying mechanisms were then investigated in the neuroblastoma cell line IMR-32. A cell viability assay showed that the IC50 values of AFB_1_ on IMR-32 cells were 6.18 μg/mL and 5.87 μg/mL after treatment for 24 h and 48 h, respectively. ROS levels in IMR-32 cells increased significantly in a time- and AFB_1_ concentration-dependent manner, which was associated with the upregulation of *NOX2*, and downregulation of *OXR1*, *SOD1*, and *SOD2*. Substantial DNA damage associated with the downregulation of *PARP1*, *BRCA2*, and *RAD51* was also observed. Furthermore, AFB_1_ significantly induced S-phase arrest, which is associated with the upregulation of *CDKN1A*, *CDKN2C*, and *CDKN2D*. Finally, AFB_1_ induced apoptosis involving *CASP3* and *BAX*. Taken together, AFB_1_ manifests a wide range of cytotoxicity on neuronal cells including ROS accumulation, DNA damage, S-phase arrest, and apoptosis—all of which are key factors for understanding the neurotoxicology of AFB_1_.

## 1. Introduction

Aflatoxin B1 (AFB_1_) is a naturally occurring mycotoxin produced by *Aspergillus* fungi that contaminates a wide range of feed and food [1]. AFB_1_ is considered the most potent naturally occurring carcinogen, with a variety of toxicities reported, including genotoxicity, mutagenicity, and immunotoxicity [2].

AFB_1_ has been reported to affect multiple organs and tissues, causing various acute and chronic diseases, most of which are severe. Since its discovery, AFB_1_ has been associated with liver cancer [3]. Epidemiological surveys reveal a strong statistical correlation between aflatoxin ingestion and incidence of hepatocellular carcinoma in several areas of the world [4,5]. In addition to its involvement in liver cancer, AFB_1_ also damages many other organs including the kidney, intestine, pancreas, bladder, bone, and viscera [6]. The toxic effects of AFB_1_ on the nervous system have been reported in several studies. Ikegwuonu showed that AFB_1_ increases the activity of β-glucuronidase and β-galactosidase in the central and peripheral nervous systems [7]. Trebak et al. showed that orally treated rat with AFB_1_ at 150 μg/kg or 300 μg/kg twice a week for 5 weeks disrupts the hypothalamic regulation of neuropeptides [8]. Baldissera showed that 1177 ppb/kg feed of AFB_1_ changes the activities of AChE and Na^+^/K^+^-ATPase in brain synaptosomes of silver catfish [9]. Makhlouf showed that the administration of 250 μg/kg B.W./day of AFB_1_ to rats for 4 weeks resulted in degeneration in the sciatic nerve in the form of Wallerian degeneration of the myelin sheath [10]. Alsayyah showed that 250 μg/kg B.W./day of AFB_1_ causes neurotoxic effects at different levels of penetration in blood-brain barrier integrity and disruption of enzymatic activity in the brain [11]. Although the toxic effects and mechanisms of AFB_1_ to the nervous system have been well reported on the histological or biochemical level, the cytotoxic effects and mechanism of AFB_1_ on neuronal cells remains unknown.

The well-known mechanism of AFB_1_ toxicity involves biological activation of the compound into a highly active epoxide (AFB_1_-8,9-exo-epoxide, AFBO) through cytochrome P450 [12]. AFBO covalently binds to DNA and proteins to form adducts that result in chronic and acute cytotoxicity. Moreover, the production of reactive oxygen species (ROS) is also an important cause of AFB_1_-induced cellular damage. Studies have shown that AFB_1_ can induce the production of ROS in multiple cell lines such as HepG2 cells, HaCaT cells, broiler chicken cardiomyocytes, and porcine oocytes [13,14,15,16]. At the cellular level, its well characterised toxic effects are apoptosis and cell cycle arrest. An intraperitoneal administration of 20 μg/kg B.W./day of AFB_1_ to mice induced overexpression of p21 with concomitant downregulation of cyclin D1 and CDK4, which inhibited the formation of cyclin-CDK complexes, ultimately leading to cell cycle arrest and apoptosis [17]. In HEK-293T cells, 1–6 μg/mL of AFB_1_ strongly induced S-phase arrest by upregulating the expression of p21 via PLK1, PLD1, and MYC [18]. For broiler chickens, 0.6 mg/kg AFB_1_ diet feeding for 3 weeks induced G2/M cell cycle arrest via the ATM pathway in the jejunum [19]. Altogether, the toxic effects and mechanisms of AFB_1_ at the cellular and molecular levels vary in different cells and organs, rendering it important to clarify the neurotoxicity of AFB_1_ at these levels.

In this study, we assessed brain damage and AFB_1_ residues in the mouse brain after intragastric AFB_1_ administration at 300 μg/kg.bw/day for 30 days, which is comparable to the contamination levels in food and feed materials, especially in developing countries [20]. Subsequently, neurotoxicity and the associated mechanisms were investigated at the cellular and molecular levels in the neuroblastoma cell line IMR-32 at concentrations near IC50 (2 or 6 μg/mL), which is commonly used in toxicological studies at cellular and molecular levels [21].

## 2. Results

### 2.1. AFB_1_ Induces Brain Damage in Mice

**Figure 1:**
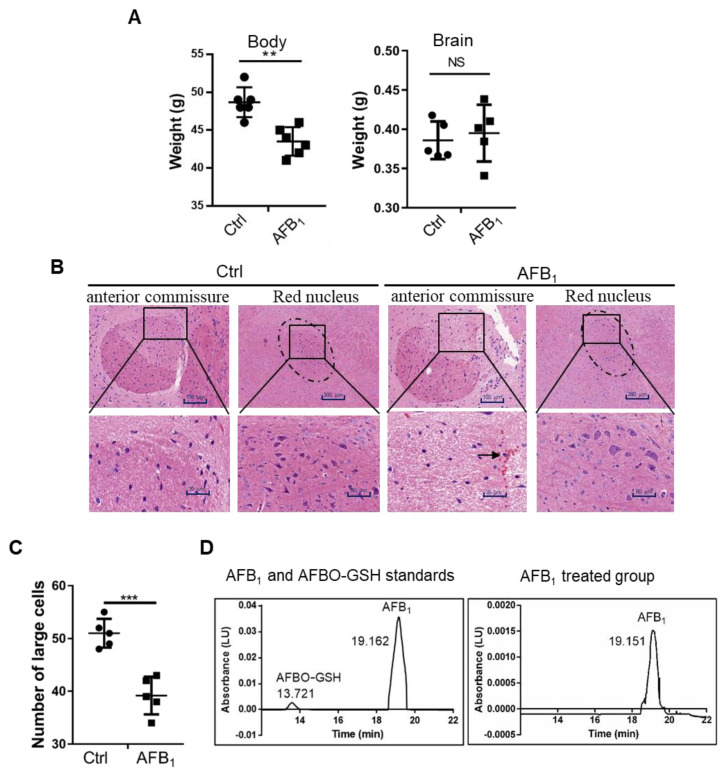
AFB_1_ enters the brain and causes brain damage after 30 days of dietary administration (300 μg/kg). (**A**) Body weight and brain weight. (**B**) Haematoxylin and eosin (HE) staining of brain histopathological sections. The edge between the anterior commissure and surrounding organisations is indicated as a square and enlarged. The dashed circle indicates red nucleus. The black arrow indicates red-cell infiltration. (**C**) The number of large cells in the red nucleus in panel B was counted. (**D**) HPLC analysis of the content of AFB_1_ in mouse brains. All experiments were performed in triplicate and the values represent the mean ± SD of three independent experiments. Statistical significance was defined as ** *p* < 0.01, or *** *p* < 0.001.

To investigate the brain toxicity of AFB_1_, 25-day-old male Kunming mice were administered intragastrically with 300 μg/kg.bw/day of AFB_1_ for 30 days. Total body weight and AFB_1_ concentration, histomorphology, and weight of the brain were then analysed. The results showed that the total body weight of mice was significantly lower in the AFB_1_-treated group, while the brain weight was not significantly affected (Figure 1A). However, pathological analysis of brain sections showed that the edges between the anterior commissure and surrounding organisations were not clear in the AFB_1_-treated group (Figure 1B). The number of large cells in the red nucleus was reduced and red-cell infiltration was evident in the brain, as indicated by the black arrow (Figure 1B,C). HPLC analysis showed that AFB_1_ accumulated in the brains of mice at approximately 60 μg/kg after AFB_1_ treatment, while no AFBO was detected (Figure 1D). Altogether, AFB_1_, but not AFBO, caused severe brain damage in mice.

### 2.2. AFB_1_ Inhibits Cell Proliferation of IMR-32 Cells

**Figure 2:**
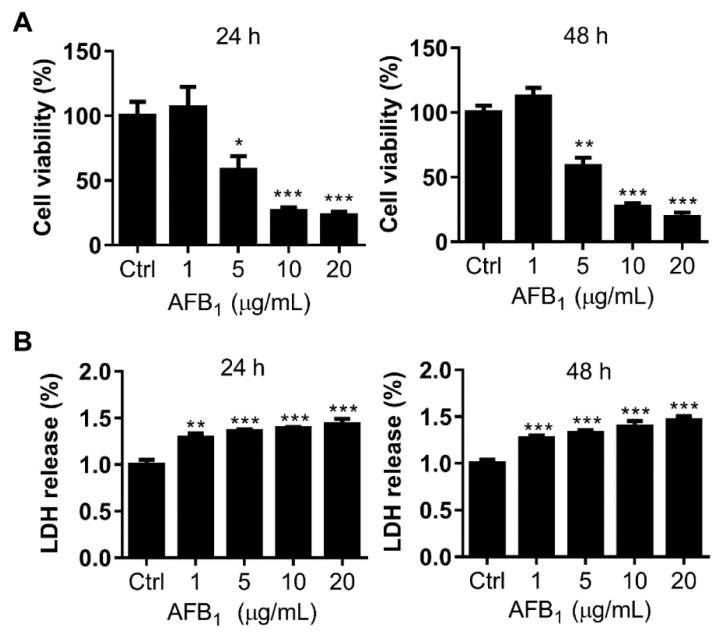
AFB_1_ exhibits significant cytotoxicity toward IMR-32 cells. (**A**) The IC50 values of AFB_1_ were 6.18 μg/mL and 5.87 μg/mL in IMR-32 cells after 24 h and 48 h of treatment, respectively. (**B**) Relative LDH release by IMR-32 cells exposed to different concentrations of AFB_1_ for 24 h and 48 h. All experiments were repeated three times and the values represent the mean ± SD of three independent experiments. Statistical significance was defined as * *p* < 0.05, ** *p* < 0.01, or *** *p* < 0.001.

To further address the molecular mechanism of the neurotoxicity of AFB_1_, the toxicity of AFB_1_ to neuroblastoma cell line IMR-32 was investigated. First, the IC50 of AFB_1_ to IMR-32 cells, as determined by 3-(4,5-dimethylthiazol-2-yl)-2,5-diphenyltetrazolium bromide (MTT) assay, was 6.18 μg/mL and 5.87 μg/mL after treatment for 24 h and 48 h, respectively (Figure 2A). Based on the calculated IC50, doses of 2 μg/mL and 6 μg/mL AFB_1_, which are below or near the IC50 values, were used on IMR-32 cells in subsequent experiments. Second, to assess the cytotoxicity of AFB_1_ to IMR-32 cells, the amount of LDH released was analyzed. The results showed that extracellular LDH increased significantly compared to the control group after treatment with 1 μg/mL, 5 μg/mL, 10 μg/mL, or 20 μg/mL AFB_1_ for 24 h or 48 h (Figure 2B), indicating that AFB_1_ disrupted the cell membrane integrity of IMR-32 cells. These results suggest that AFB_1_ is toxic to IMR-32 cells with an IC50 of approximately 6 μg/mL.

### 2.3. AFB_1_ Enhances Intracellular ROS Levels

**Figure 3:**
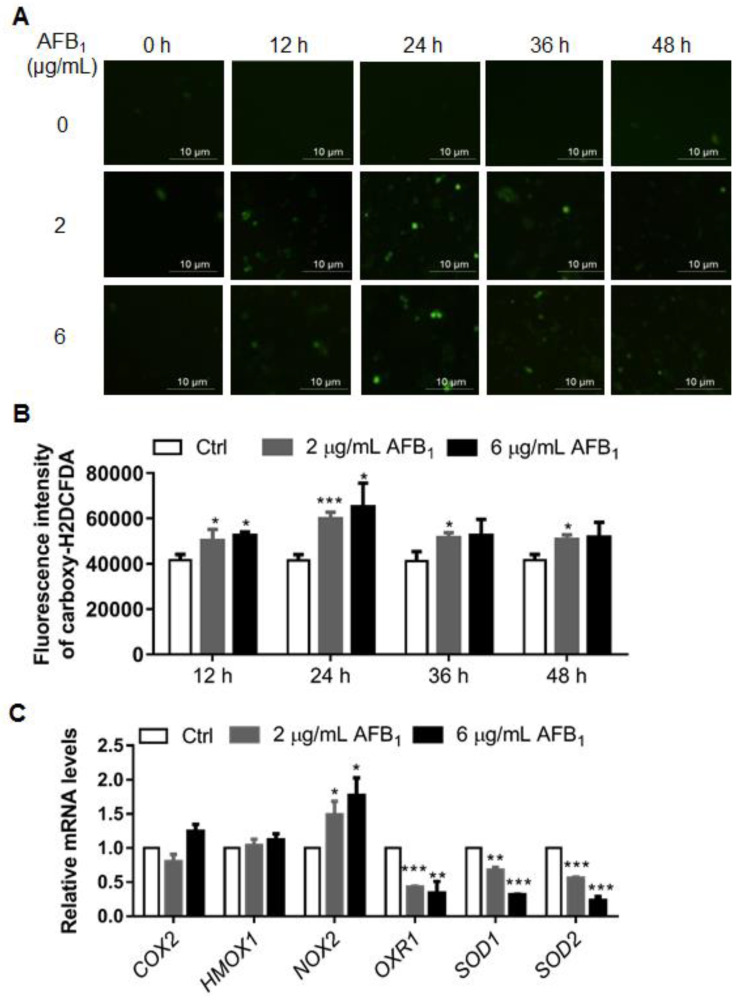
AFB_1_ enhances intracellular ROS levels and inhibits the transcription of antioxidative stress-associated genes in IMR-32 cells. (**A**) ROS levels were detected by a carboxy-H2DCFDA probe (Green colour) and observed under a fluorescence microscope. (**B**) ROS production in IMR-32 cells after AFB_1_ exposure was analyzed by flow cytometry after staining with carboxy-H2DCFDA; the data were analyzed using Flow Jo software (BD, Franklin Lakes, USA). (**C**) The mRNA levels of ROS-related genes in IMR-32 cells were analyzed using qRT-PCR after exposure to 2 μg/mL or 6 μg/mL AFB_1_. All the mRNA levels tested were normalized using the mRNA levels of *GAPDH* as the internal control. All experiments were performed in triplicate and the values represent the mean ± SD of three independent experiments. Statistical significance was defined as * *p* < 0.05, ** *p* < 0.01, or *** *p* < 0.001.

The accumulation of reactive oxygen species (ROS) under AFB_1_ treatment has been widely reported in several cell lines [14]. Therefore, we next investigated the ROS levels of IMR-32 cells under AFB_1_ treatment. The IMR-32 cells were treated with 2 μg/mL or 6 μg/mL of AFB_1_ for 12 h, 24 h, 36 h, and 48 h. Then, the ROS was stained by a carboxy-H2DCFDA probe, observed by fluorescence microscopy, and qualified by flow cytometry. The intracellular ROS levels were significantly enhanced compared to those in the control group after 12 h of treatment, reaching their highest levels after 24 h treatment and recovering to a steady level at 36 h (Figure 3A,B). To investigate the mechanism by which AFB_1_ upregulates ROS in IMR-32 cells, the expression of six genes, *COX2*, *HOMX1*, *NOX2*, *OXR1*, *SOD1*, and *SOD2*, which are involved in ROS production and metabolism, was investigated. The mRNA levels of *COX2* and *HOMX1* have no significant changes under AFB_1_ treatment in IMR-32 cells (Figure 3C). The mRNA levels of *NOX2*, a gene associated with ROS production, were upregulated 1.51- and 1.74-fold, respectively, in IMR-32 cells treated with 2 µg/mL and 6 µg/mL AFB_1_ for 24 h (Figure 3C). The mRNA levels of three antioxidative genes—*OXR1*, *SOD1*, and *SOD2*—were downregulated 0.42-, 0.67-, and 0.56-fold, respectively, in IMR-32 cells treated with 2 µg/mL AFB_1_ for 24 h (Figure 3C). At 6 µg/mL AFB_1_ treatment for 24 h, the expression levels of *OXR1*, *SOD1*, and *SOD2* were further downregulated, with fold changes of 0.34-, 0.31-, and 0.23-fold, respectively (Figure 3C). Collectively, these results show that AFB_1_ induced ROS accumulation in IMR-32 cells and was associated with a ROS production gene—*NOX2*, and three antioxidative genes—*OXR1*, *SOD1*, and *SOD2*.

### 2.4. AFB_1_ Induces DNA Damage

**Figure 4:**
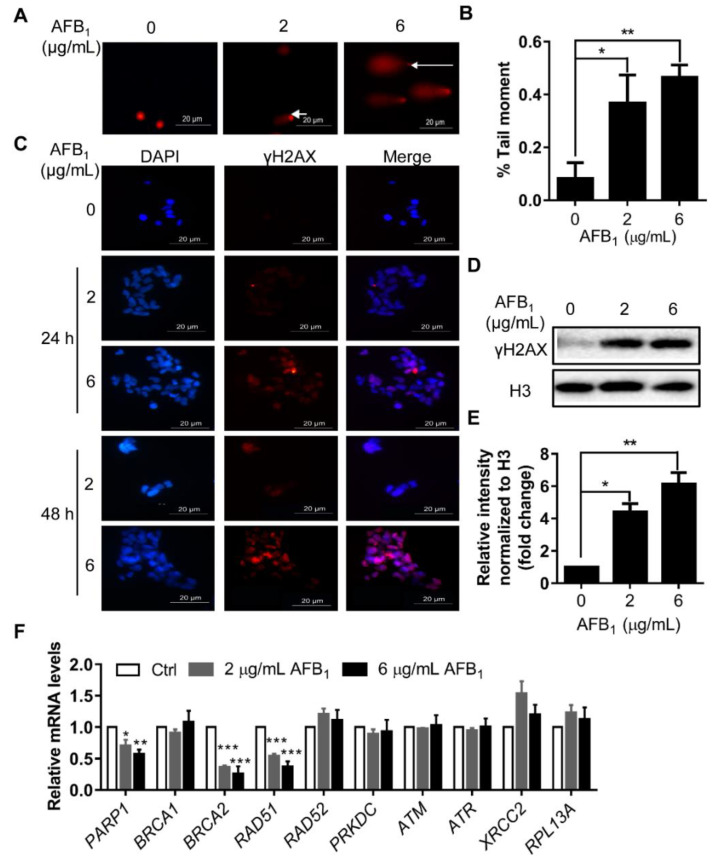
AFB_1_ induces DNA damage and inhibits transcription of DNA damage response genes in IMR-32 cells. (**A**) A comet assay was used to detect DNA damage after treatment with different concentrations of AFB_1_ (2 μg/mL and 6 μg/mL) for 24 h. White arrows indicate tail DNA. (**B**) Quantification of the tail moment in panel A by ImageJ. (**C**) Representative immunofluorescence staining image of γH2AX in IMR-32 cells after 2 or 6 μg/mL AFB_1_ treatment for 24 h and 48 h. Blue colour: nucleus; Red colour: γH2AX; (**D**) Representative Western blot of γH2AX in IMR-32 cells after treatment with different concentrations of AFB_1_ (2 μg/mL and 6 μg/mL) for 24 h. (**E**) Quantification of the fold change of γH2AX protein shown in panel D. (**F**) Fold changes in the mRNA levels of DNA repair-related genes in IMR-32 cells after 24 h of exposure to 2 μg/mL or 6 μg/mL AFB_1_. All the mRNA levels tested were normalized to *GAPDH*. All experiments were performed in triplicate and the values represent the mean ± SD of three independent experiments. Statistical significance was defined as * *p* < 0.05, ** *p* < 0.01, or *** *p* < 0.001.

ROS levels are usually correlated with DNA damage. The DNA damage of IMR-32 cells under AFB_1_ treatment was investigated by comet assay. Tail DNA was quantified by ImageJ. The results showed that the quantity of tail DNA after treatment with 2 µg/mL and 6 μg/mL AFB_1_ for 24 h was upregulated by 3.6- and 4.7-fold, respectively (Figure 4A,B). Subsequently, the DNA damage marker γH2AX was detected by immunofluorescence. The γH2AX levels in IMR-32 cells were significantly upregulated after treatment with 2 µg/mL and 6 μg/mL AFB_1_ for 24 h and 48 h (Figure 4C–E). After treatment with 2 µg/mL and 6 μg/mL AFB_1_ for 24 h, the levels of γH2AX increased by 4.5- and 6.1-fold, respectively (Figure 4D,E). To further investigate the mechanism by which AFB_1_ induces DNA damage, the mRNA levels of genes involved in DNA damage response were analyzed. The results showed that the mRNA levels of *PARP1*, *BRCA2*, and *RAD51* were significantly downregulated under AFB_1_ treatment, whereas *RAD52*, *PRKDC*, *ATM*, *ATR*, *XRCC2*, and *RPL13A* did not change (Figure 4F). After treatment with 2 μg/mL AFB_1_ for 24 h, the mRNA levels of *PARP1*, *BRCA1*, *BRCA2*, and *RAD51* were downregulated by 0.70-, 0.35-, and 0.53-fold, respectively. After treatment with 6 μg/mL AFB_1_ for 24 h, the mRNA levels of *PARP1*, *BRCA2*, and *RAD51* were downregulated by 0.56-, 0.25-, and 0.37-fold, respectively (Figure 4F). Taken together, these results indicate that AFB_1_ significantly induced DNA damage in IMR-32 cells and three DNA damage response genes—*PARP1*, *BRCA2*, and *RAD51*—were involved in this process.

### 2.5. AFB_1_ Induces S-Phase Cell Cycle Arrest

**Figure 5:**
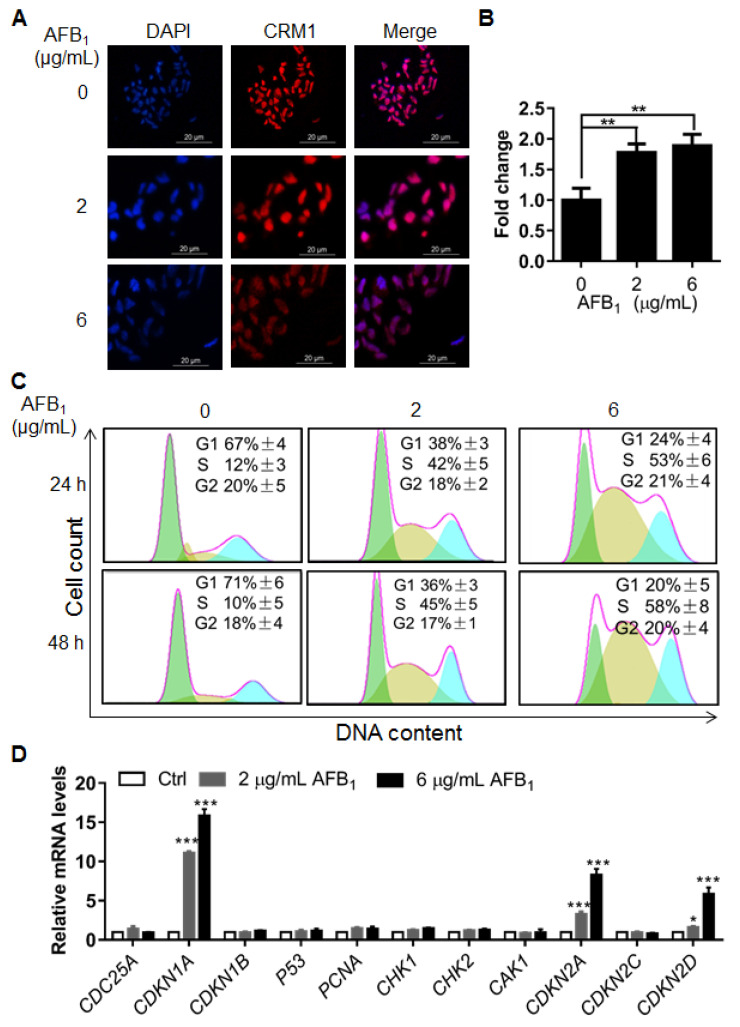
The cell cycle is arrested in S phase upon treatment with AFB_1_ in IMR-32 cells. (**A**) Representative immunofluorescence staining images of the nuclei of IMR-32 cells after 2 and 6 μg/mL AFB_1_ treatment for 24 h. CRM1 was used to determine nuclear size. Blue colour: nucleus; Red colour: CRM1; (**B**) Quantification of nuclear size in panel A by ImageJ. (**C**) Cell cycle analysis of IMR-32 cells by flow cytometry following AFB_1_ treatment. The data were analyzed with FlowJo software. (**D**) Fold changes in the mRNA levels of cell cycle-related genes in IMR-32 cells after exposure to 2 μg/mL or 6 μg/mL AFB_1_ for 24 h. All of the mRNA levels tested were normalized to *GAPDH*. All experiments were performed in triplicate and the values represent the mean ± SD of three independent experiments. Statistical significance was defined as * *p* < 0.05, ** *p* < 0.01, or *** *p* < 0.001.

To further investigate the cellular damage caused by AFB_1_, nuclear size was determined by immunofluorescence using an antibody (anti-CRM1) against the nuclear membrane and DAPI staining. Interestingly, nuclear size increased significantly after treatment with 2 and 6 μg/mL of AFB_1_ (Figure 5A,B), indicating that AFB_1_ might induce other abnormalities in DNA, e.g., polyploidy. Next, the cell cycle of IMR-32 cells under treatment with AFB_1_ was analyzed by flow cytometry. The results showed that AFB_1_ induced significant S-phase arrest at concentrations of 2 µg/mL and 6 μg/mL after treatment for 24 h or 48 h (Figure 5C). To further investigate the mechanism of AFB_1_-induced S-phase arrest, the mRNA levels of cell cycle-related genes of IMR-32 cells after AFB_1_ treatment were analyzed by qRT-PCR. The results showed that the mRNA levels of *CDKN1A*, *CDKN2C* and *CDKN2D* were upregulated by 10.81-, 3.32-, and 1.61-fold after treatment with 2 μg/mL AFB_1_ for 24 h. At 6 μg/mL AFB_1_ treatment, the mRNA levels of *CDKN1A*, *CDKN2C* and *CDKN2D* were upregulated by 15.83-, 8.31-, and 5.86-fold, respectively (Figure 5D). Altogether, these results show that AFB_1_ significantly increased nuclear size and induced significant S-phase cell cycle arrest in IMR-32 cells, which was associated with the upregulation of *CDKN1A*, *CDKN2C* and *CDKN2D*.

### 2.6. AFB_1_ Induces Apoptosis

**Figure 6:**
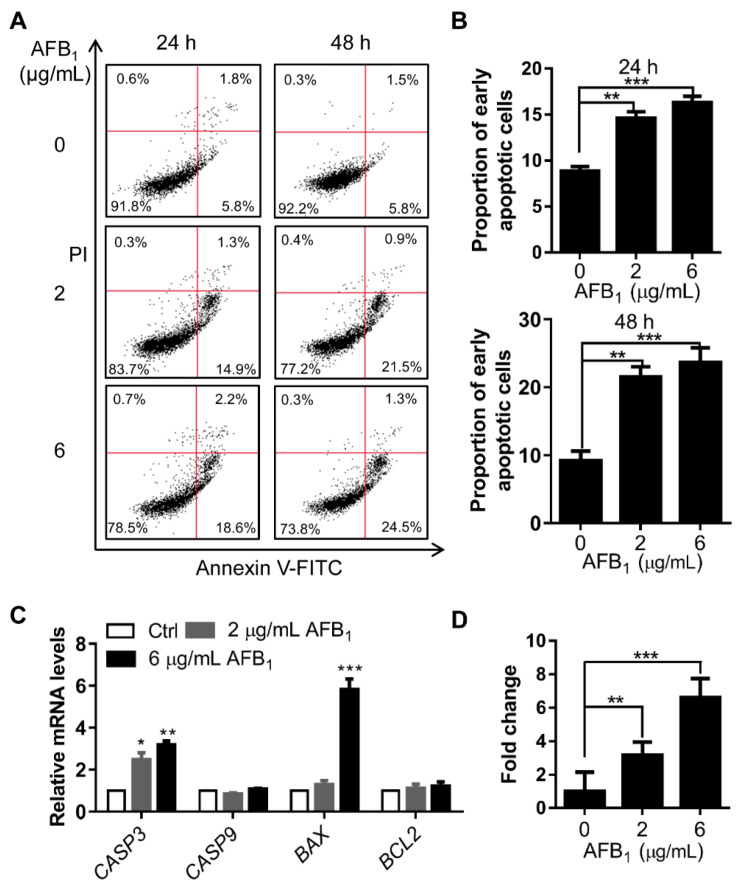
AFB_1_ induces apoptosis in IMR-32 cells. (**A**) Apoptosis analysis of IMR-32 cells after AFB_1_ treatment by flow cytometry. The data were analyzed with FlowJo software. (**B**) The proportion of early apoptotic cells was calculated for the cells in panel A. (**C**) Fold changes in the mRNA levels of apoptosis-related genes in IMR-32 cells after exposure to 2 μg/mL or 6 μg/mL AFB_1_ for 24 h. All the mRNA levels tested were normalized to *GAPDH*. (**D**) The enzyme activity of caspase-3 in cells was detected via ELISA after treatment with 2 μg/mL or 6 μg/mL AFB_1_ for 24 h. All experiments were performed in triplicate and the values represent the mean ± SD of three independent experiments. Statistical significance was defined as * *p* < 0.05, ** *p* < 0.01, or *** *p* < 0.001.

DNA damage is directly related to apoptosis. Apoptosis in IMR-32 cells treated with AFB_1_ was analyzed by flow cytometry. After treatment with 2 μg/mL and 6 μg/mL AFB_1_, the early apoptotic rates of IMR-32 cells were upregulated 1.7- and 1.8-fold, respectively, after 24 h of treatment and 2.4- and 2.7-fold, respectively, after 48 h of treatment (Figure 6A, B). To investigate the mechanism by which AFB_1_ induced apoptosis in IMR-32 cells, the mRNA levels of genes associated with apoptosis were examined. The results showed that the mRNA levels of *CASP3* were significantly upregulated 2.4- and 3.18-fold after 2 μg/mL and 6 μg/mL AFB_1_ treatment, respectively, and the mRNA levels of *BAX* were significantly upregulated 5.82-fold under 6 μg/mL AFB_1_ treatment (Figure 6C). Subsequently, we determined caspase-3 activity in cells after AFB_1_ treatment via ELISA. The activity of caspase-3 was upregulated 3.0- and 6.3-fold after AFB_1_ treatment (Figure 6D). Altogether, these results suggested that AFB_1_ induced significant apoptosis and that caspase-3 and BAX were involved in this process.

## 3. Discussion

Up to 4.5 billion humans are believed to be at risk of exposure to aflatoxins, which are known to have deleterious effects on the health of both humans and animals [22]. Multiple toxicities and mechanisms have been reported, especially carcinogenicity. In this study, we found that AFB_1_ induced brain damage in mice. Mechanistically, AFB_1_ induces accumulation of ROS, DNA damage, S-phase arrest, and apoptosis.

To date, several toxic effects and mechanisms of AFB_1_ to the nervous system have been reported. The present study showed that after AFB_1_ treatment in mice, the edges of the anterior part of the anterior commissure were not clear in the AFB_1_-treated group (Figure 1B). Moreover, the number of large cells in the red nucleus was reduced and red cell infiltration was evident in the brain (Figure 1B,C). The anterior commissure of the brain plays a crucial function in bodily behaviour and damage to the anterior commissure affects a variety of such activities [23]. The red nucleus of the brain is composed of large and small cells, both of which are important for the regulation of body posture and maintenance of exercise coordination [24,25]. Previous reports showed that AFBO is the main toxic metabolite attributed to multiple toxicities [12]. Interestingly, no AFBO residues were detected in brain tissue extract (Figure 1D), indicating that AFB_1_ might not exert its toxicological effects in the brain through AFBO. Altogether, AFB_1_ is highly toxic to the nervous system.

To elucidate the mechanisms of neurotoxicity caused by AFB_1_, the toxic effects and associated mechanism of AFB_1_ were studied in IMR-32 cells. AFB_1_ inhibited the proliferation and impaired the integrity of IMR-32 cells in a concentration- and time-dependent manner (Figure 2). Previous studies have shown that AFB_1_ induces ROS production in several cell lines and is considered a main mechanism [26,27,28]. In this study, intracellular ROS levels were also significantly upregulated in IMR-32 cells after AFB_1_ treatment for 12 h or 24 h and the mRNA levels of antioxidative enzymes were downregulated (Figure 3). Similarly, previous studies have shown that antioxidative enzyme levels were significantly reduced in hepatocytes after AFB_1_ treatment [29]. Consistent with this assumption, the decrease in antioxidative enzyme levels may constitute a crucial factor causing the accumulation of ROS in cells. Accumulation of intracellular ROS usually leads to DNA damage [30,31]. DNA damage was significantly enhanced in IMR-32 cells after treatment with AFB_1_ (Figure 4). The expression levels of the DNA damage response genes *PARP1*, *BRCA2*, and *RAD51* were also significantly downregulated after AFB_1_ treatment (Figure 4). Suppression of DNA repair-gene expression inhibits DNA damage repair and promotes the DNA damage process [32]. These results suggest that AFB_1_ induces DNA damage by inducing intracellular ROS production and suppresses the DNA damage-response genes *PARP1*, *BRCA2*, and *RAD51*.

DNA damage usually leads to cell cycle arrest and apoptosis [33,34]. In different cells, AFB_1_ can induce cell cycle arrest at different phases. For example, previous studies showed that AFB_1_ treatment can induce G0/G1 arrest in F344 rat livers [35] and S-phase arrest in HepG2 cells [36]. In this study, significant increases in nuclear size were observed after AFB_1_ treatment, which signify increases in intracellular DNA content and abnormal cell division [37]. AFB_1_ significantly induced S-phase arrest in IMR-32 cells as further evidenced by the upregulation of the cell cycle regulatory genes *CDKN1A*, *CDKN2C*, and *CDKN2D* after 2 μg/mL and 6 μg/mL AFB_1_ treatment (Figure 5). Upregulation of *CDKN1A* and *CDKN2C* in cells has previously been shown to lead to S-phase arrest [38,39]. Therefore, we concluded that the upregulation of *CDKN1A*, *CDKN2C*, and *CDKN2D* by AFB_1_ may be involved in the induction of S-phase arrest. In addition, DNA damage can induce apoptosis [40]. In our study, the early apoptotic rate increased significantly after AFB_1_ treatment. There are many apoptosis-activating pathways in cells, mainly the caspase 3, 6, 7, and 9 pathways; certain studies have shown that upregulation of *BAX* can also induce apoptosis [41,42,43]. A previous study reported that caspase-3/7 was activated by AFB_1_ in mammalian leukocytes [44]. In this study, the transcription levels of *CASP3* and *BAX* were upregulated and those of activated caspase-3 were significantly upregulated in cells after 2 μg/mL and 6 μg/mL AFB_1_ treatment (Figure 6). BAX is involved in mitochondrial pathway-mediated apoptosis [45] and caspase-3 is a cysteine protease involved in the “execution” phase of cellular apoptosis [46]. Altogether, these results suggest that AFB_1_ induces the upregulation of the cell-cycle regulatory genes *CDKN1A*, *CDKN2C*, and *CDKN2D* and proapoptotic genes *CASP3* and *BAX*, thus mediating S-phase arrest and apoptosis.

In summary, we found that AFB_1_ induces neuronal damage in a multifaceted manner with neuro-cytotoxicity. AFB_1_ damages mouse neural tissue and induces intracellular ROS accumulation, DNA damage, S-phase arrest, and apoptosis in IMR-32 cells.

## 4. Materials and Methods

### 4.1. Animals and Cells

Male Chinese Kunming (KM) mice and the standard diets used in the study were purchased from Guangdong Medical Laboratory Animal Centre (Guangzhou, China). The mice were 25 days old and approximately 14–16 g in weight at the beginning of the experiment. We used male mice as subjects because they are more sensitive to AFB_1_ than female mice [47]. The mice were kept under standard laboratory conditions (temperature 25–28 °C with a 12-h light/12-h dark cycle) in the Laboratory Animal Centre of the South China Agricultural University. Food and water were available ad libitum. All experiments were approved by the Animal Care Committee of the South China Agricultural University on April 30th, 2018 (Permit No. 2018037) and carried out in strict accordance with the regulations of the Administration of Affairs Concerning Experimental Animals of Guangdong Province, China. The 10 mice were divided into two groups: control group (PBS) and AFB_1_ treatment group (*n* = 5). AFB_1_ (Pribolab, Qingdao, China) was dissolved in DMSO at a concentration of 10 mg/mL. The treatment group was administered AFB_1_ by gavage once a day at a dose of 300 μg/kg/day for 30 days [48]. At the end of treatment, the mice were anesthetized and sacrificed by cervical dislocation. The cranial cavity was opened and brain tissues were harvested according to a protocol described previously [49]. The brain samples were fixed in 4% paraformaldehyde and sent to Google Biology (Wuhan, China) for pathological sectioning. The IMR-32 cell line used in this experiment is maintained in our laboratory and was cultured in MEM (Gibco, Waltham, MA, USA) with 10% FBS (Biological Industries, Beit Haemek, Israel).

### 4.2. HPLC Analysis of AFB_1_ and AFBO

AFB_1_ and its metabolites in brain tissue were extracted and analyzed as previously reported [18]. Briefly, brain tissue samples were ground into powder and extracted by 70% methanol. The content of AFB_1_ and AFBO in the extract was analyzed by HPLC and calculated based on the peak areas.

### 4.3. Cell Viability Assay and LDH Release Assay

The cytotoxicity of AFB_1_ to IMR-32 cells was measured using MTT and LDH release assays as previously described [18]. Briefly, IMR-32 cells were seeded into 96-well plates and treated with AFB_1_ at concentrations ranging from 0–10 μg/mL for 24 h or 48 h. Then, the cell viability was measured using MTT and LDH release assays. All experiments were repeated three times independently.

### 4.4. RNA Extraction and qRT-PCR

IMR-32 cells were cultured in 6-well culture plates and treated with 2 μg/mL or 6 μg/mL AFB_1_. The total RNA was extracted using TRIzol solution (Invitrogen, Carlsbad, CA, USA) according to the manufacturer’s instructions. RNA was transcribed into cDNA using a Prime Script™ RT Reagent Kit (Takara, Taichung, Japan). qRT-PCR was performed on a Bio-Rad CFX96 real-time PCR detection system (Bio-Rad, Hercules, CA, USA) according to the manufacturer’s recommendations. mRNA expression levels were measured using qRT-PCR in 20 μL volumes containing SYBR Green I Dye (Promega, Madison, WI, USA). The primers used for qRT-PCR are listed in Table S1. The 2^−ΔΔCT^ method was employed to determine the relative expression levels of the target genes normalized to *GAPDH*, and the experiments were repeated three times independently.

### 4.5. Western Blot

IMR-32 cells were treated with 2 μg/mL or 6 μg/mL AFB_1_ for 24 h. The cells were harvested and analyzed by Western blot as previously reported [18]. The primary antibodies against γH2AX (Cat. No. 5438s) and H3 (Cat. No. 4499s) and secondary antibody were purchased from Cell Signaling Technology (Danvers, MA, USA).

### 4.6. Immunofluorescence

IMR-32 cells after treatment with different concentrations of AFB_1_ were fixed in 4% paraformaldehyde at 25 °C for 20 min, then permeabilized in 0.5% Triton X-100 in PBS for 10 min. The slides were incubated with primary antibodies against nuclear membrane protein CRM1 (Cat. No. 46249s, Cell Signaling Technology, Danvers, MA, USA) or γH2AX (Cat. No. 80,312 Cell Signaling Technology, Danvers, MA, USA), and then incubated with secondary antibody conjugated to Alexa Fluor 546 (Cat. No. ab60317, Abcam, Cambridge, UK). The nuclei were stained with DAPI (Sigma-Aldrich, St. Louis, MO, USA). The images were observed with an Olympus IX71 microscope (Olympus, Tokyo, Japan).

### 4.7. Comet Assay

IMR-32 cells were trypsinised after exposure to 2 μg/mL and 6 μg/mL AFB_1_ for 24 h, collected in growth medium, centrifuged for 5 min at 300× *g* and suspended in PBS. For single-cell gel electrophoresis, cells were embedded in 0.5% low-melting point agarose. The cells were lysed overnight at 4 °C in lysis buffer (2.5 M NaCl, 100 mM EDTA, 10 mM Tris Base, 1% sodium lauroyl sarcosinate, and 1% Triton X-100, pH 10) and placed in an alkaline solution (0.3 M NaOH and 1 mM EDTA, pH > 13) for 25 min before being subjected to electrophoresis (25 min at 25 V and 300 mA). The DNA was visualised with ethidium bromide (10 μg/mL) (Dingguo, Beijing, China), and the comets were observed with an Olympus IX71 microscope (Olympus, Tokyo, Japan) and analyzed with ImageJ software (National Institutes of Health, Bethesda, MD, USA). Fifty randomly selected cells on each slide were scored and the percentage of DNA in the tail was used as the endpoint. To create the figures, we used the median tail intensity from four independent experiments.

### 4.8. Cell Cycle, ROS, and Apoptosis Analysis

IMR-32 cells were exposed to AFB_1_ at one of two concentrations (2 μg/mL or 6 μg/mL) or to 0.01% DMSO as a negative control for 24 h or 48 h. The cells were washed with precooled PBS after digestion with 0.25% trypsin (Gibco, Grand Island, NY, USA) and fixed with 70% ethanol at 4 °C overnight. Then, the cells were washed with precooled PBS and collected by centrifugation. The cells were resuspended with 500 μL of PI staining reagent (Dingguo, Beijing, China) and incubated for 30 min at 37 °C. Cell cycle was measured with flow cytometry (BD, Franklin Lakes, SA, USA) and the data were analyzed with Flow Jo™ software (BD, Franklin Lakes, NJ, USA).

To evaluate the effect of AFB_1_ on ROS levels, cells treated with AFB_1_ were collected by trypsinisation, washed twice with ice-cold PBS, then suspended in PBS at a concentration of approximately 1 × 10^6^ cells/mL. The suspensions were then briefly vortexed and incubated with 10 μM carboxy-H2DCFDA in the dark at 37 °C for 30 min. After incubation, the samples were analyzed with an Accuri C6 Flow Cytometer (BD, Franklin Lakes, NJ, USA). Simultaneously, sample fluorescence signals were observed under a fluorescence microscope (Olympus, Tokyo, Japan). All experiments were repeated three times independently.

To determine the proportion of apoptotic cells using an Annexin V-FITC/PI kit (BD, Franklin Lakes, NJ, USA), the cells were first seeded in 6-well cell culture plates then harvested after treatment with AFB_1_ for 24 h or 48 h. The culture medium was discarded and the cells were digested with 0.25% trypsin and washed twice with precooled PBS. Next, the cells were resuspended in 1× binding buffer and 100 μL of cell solution was transferred to a new tube. Five microliters each of the Annexin V-FITC and PI solutions were added to each cell solution. The cells were gently vortexed and incubated for 15 min at 25 °C in the dark, and 400 μL of 1× binding buffer was added to each sample. Apoptosis was measured via flow cytometry (BD, Franklin Lakes, NJ, USA).

### 4.9. Caspase-3 Activity Assay

To investigate the mechanism of AFB_1_-mediated apoptosis, the activity of caspase-3 was determined with a caspase-3 spectrophotometric test kit (Wanleibio, Shenyang, China). IMR-32 cells were treated with 2 μg/mL or 6 μg/mL of AFB_1_ for 24 h. At the end of treatment, the cells were washed once with PBS and lysed with 100 μL of cell lysis buffer from the kit. Ten microliters each of detection buffer and Ac-DEVD-pNA were added to the supernatant after centrifugation. The supernatant was incubated at 37 °C for 2–4 h and the absorbance at 490 nm was measured with a multimode plate reader (Molecular Devices, San Francisco, CA, USA). Caspase-3 activity was calculated based on the standard curve. All experiments were performed in triplicate independently.

### 4.10. Statistical Analysis

All statistical analyses were performed using SPSS Version 16.0 (SPSS Inc., Chicago, IL, USA). Statistically significant differences among more than two groups were determined using one-way analysis of variance (ANOVA) followed by Bonferroni’s multiple comparison tests. Significance was defined as *** *p* < 0.001, ** *p* < 0.01, and * *p* < 0.05.

## Supplementary Materials

Supplementary materials can be found at https://www.mdpi.com/1422-0067/21/18/6517/s1. Table S1. The primers used in qRT-PCR.
