# Supplementary material for: Aflatoxin B1 Induces Neurotoxicity through Reactive Oxygen Species Generation, DNA Damage, Apoptosis, and S-Phase Cell Cycle Arrest
Source: Int J Mol Sci. 2020 Sep 6;21(18):6517. doi: 10.3390/ijms21186517 (PMC7554769; doi:10.3390/ijms21186517)
Supplement: Supplementary file 1 [file ijms-21-06517-s001.pdf]

Supplementary data

Table S1. The primers used in qRT-PCR.

Gene name	Forward primer（5′-3′）	Reverse primer（5′-3′）
COX2	CTGGGAAGCCTTCTCTAACC	ACTGATGCGTGAAGTGCTGG
HMOX1	CTTTGAGGAGTTGCAGGAGC	TAAGGACCCATCGGAGAAGC
NOX2	GGCACACATTCACACTGACC	GCATTGTTCCTTTCCTGCAT
OXR1	TGTCCGGAGTTTGAGGTCTT	GATCTCCATCAAGCCAAAGC
SOD1	AGATGACTTGGGCAAAGGTG	TATTGGGCGATCCCAATTA
SOD2	CTGATTTGGACAAGCAGCAA	CTGGACAAACCTCAGCCCTA
PARP1	GGCAGCCTGATGTTGAGGT	GCGTACTCCGCTAAAAAGTCAC
BRCA1	ATCCTCTCAGAGTGACATTTTA	TTATCAGGTTATGTTGCATGGT
BRCA2	ATGCTTCAGAGCCACACACAG	TCATTTGGGTTGATCCAGGTA
RAD51	CAATGCAGATGCAGCTTGAA	CCTTGGCTTCACTAATTCCCT
RAD52	CTGCGCGGCCTCCATCTAA	GATTCTGGTTGACCTGCGCC
PRKDC	AAAATTGGTACCCCCGACAG	TTCTCATGACCCAGGAGTAGC
ATM	TGCCAGACAGCCGTGACTTAC	ACCTCCACCTGCTCATACACAAG
ATR	GGGATGCCACTGCTTGTTATGAC	CTGTCCACTCGGACCTGTTAGC
XRCC2	CCATCAGTGACAGACTGGATTA	TCTTGACCTCCTGAGCG
RPL13A	AAGGTGTTTGACGGCAT	CTCTTCTCCTCCAGGGT
LMNB2	CCTGGACATGGAGATCAACGC	CAGGTCGATCTCCTCGATGC
SYNE2	TGCCAGGGCTCTAAGACACG	TTGGATCGGTGACATGAGCC
NUP188	CTTTCGTTCTGACCTCGTTGG	GCTGTGGACTTCCCTGATACC
CDC25A	CAACCACTGGAGGTGAAGAACA	CCCAACAGCTTCTGAGGTAGGG
CDKN1A	CTGCCCAAGCTCTACCTTCC	TGGTCTTCCTCTGCTGTCCC
CDKN1B	ACGCCAGACGTAAACAGCTC	TCCAATGCTTTTAGAGGCAGA
P53	ATCCTTACCATCATCACACTGGA	CAGGACAGGCACAAATACGAAC
PCNA	GCCCTCAAAGACCTCATCAA	TCTGGGATTCCAAGTTGCTC
CHK1	CGATTCTGCTCCTCTAGCTCTGC	TGACACACCACCTGAAGTGACT
CHK2	CAGGTTCTAGCCCAGCCTTCTAC	GGAGTTCACAACACAGCAGCAC
CAK1	AAGCGTTATGAGAAGCTGGA	CCAAAAGCATCAAGGAGACC
CDKN2A	GGAGTTTTCAGAAGGGGTTTGT	CCTCATTCCTCTTCCTTGGTTT
CDKN2C	GGGGACCTAGAGCAACTTAC	CGAAACCAGTTCGGTCTTTC
CDKN2D	CTCCACTAGGACCTTCAGGG	CATGCTGCTGGAGGAGG
CASP3	AACTGGACTGTGGCATTGAG	AATAACCAGGTGCTGTGGAG
CASP9	CGAACTAACAGGCAAGCAGC	CCGACATCACCAAATCCTCC
BAX	GGGTGGTTGCCCTTTTCTACT	CCCGGAGGAAGTCCAGTGTC
BCL2	ATGGGGTGAACTGGGGGATTG	TTCCGAATTTGTTTGGGGCAGGT
GAPDH	AACGGATTTGGTCGTATTGG	GATTTTGGAGGGATCTCGC
